# Structural Basis for Acyl Acceptor Specificity in the Achromobactin Biosynthetic Enzyme AcsD

**DOI:** 10.1016/j.jmb.2011.07.059

**Published:** 2011-09-23

**Authors:** Stefan Schmelz, Catherine H. Botting, Lijiang Song, Nadia F. Kadi, Gregory L. Challis, James H. Naismith

**Affiliations:** 1 Scottish Structural Proteomics Facility and Centre for Biomolecular Sciences, The University of St Andrews, Scotland KY16 9ST, UK; 2 Department of Chemistry, University of Warwick, Coventry CV4 7AL, UK

**Keywords:** AcsD, achromobactin biosynthesis protein D, NRPS, non-ribosomal peptide synthetase, NIS, NRPS-independent siderophore, MS, mass spectrometry, HRMS, high-resolution mass spectrometry, MS/MS, tandem mass spectrometry, CEDA, (3′*S*)-*N*-citryl-ethylenediamine, PDB, Protein Data Bank, siderophores, mechanism, mass spectrometry, crystallography, chemical biology

## Abstract

Siderophores are known virulence factors, and their biosynthesis is a target for new antibacterial agents. A non-ribosomal peptide synthetase-independent siderophore biosynthetic pathway in *Dickeya dadantii* is responsible for production of the siderophore achromobactin. The *D. dadantii* achromobactin biosynthesis protein D (AcsD) enzyme has been shown to enantioselectively esterify citric acid with l-serine in the first committed step of achromobactin biosynthesis. The reaction occurs in two steps: stereospecific activation of citric acid by adenylation, followed by attack of the enzyme-bound citryl adenylate by l-serine to produce the homochiral ester. We now report a detailed characterization of the substrate profile and mechanism of the second (acyl transfer) step of AcsD enzyme. We demonstrate that the enzyme catalyzes formation of not only esters but also amides from the citryl-adenylate intermediate. We have rationalized the substrate utilization profile for the acylation reaction by determining the first X-ray crystal structure of a product complex for this enzyme class. We have identified the residues that are important for both recognition of l-serine and catalysis of ester formation. Our hypotheses were tested by biochemical analysis of various mutants, one of which shows a reversal of specificity from the wild type with respect to non-natural substrates. This change can be rationalized on the basis of our structural data. That this change in specificity is accompanied by no loss in activity suggests that AcsD and other members of the non-ribosomal peptide synthetase-independent siderophore superfamily may have biotransformation potential.

## Graphical Abstract

**Figure:**
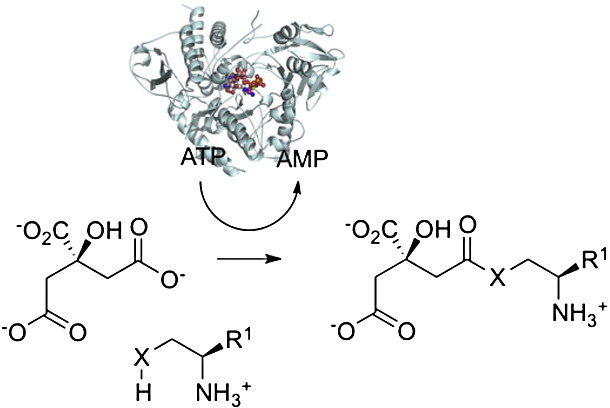

**Highlights:**

► Structure of AcsD with product. ► Description of enzyme tolerance for various nucleophiles. ► Mutagenesis of enzyme to alter substrate preference.

## Introduction

**Fig. 1:**
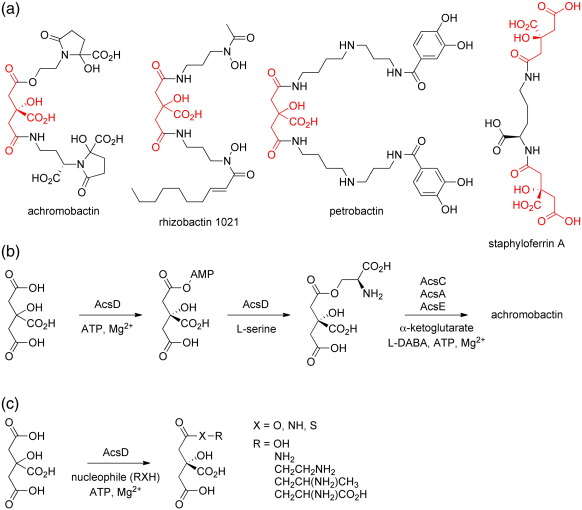
(a) Siderophores biosynthesized by prokaryotes that contain one or more citric-acid-derived building blocks (highlighted in red). (b) Proposed pathway for achromobactin biosynthesis highlighting the reactions catalyzed by AcsD. (c) Reactions of citric acid with l-serine and analogues catalyzed by AcsD, resulting in amides and ester (and thioester) derivatives of citric acid.

Citric acid plays a key role in vital metabolic pathways such as in the Krebs cycle. In addition, several microorganisms make use of citric acid as a building block in the biosynthesis of siderophores, including achromobactin,^1^ rhizobactin 1021,^2^ staphyloferrin A,^3^ and petrobactin^4^ (Fig. 1a). Siderophores are common virulence factors in bacteria, which use them to sequester iron from the host or the environment.^5^ In bacteria, siderophores are predominantly biosynthesized via two pathways:^6^ a non-ribosomal peptide synthetase (NRPS)-dependent route^7^ and an NRPS-independent siderophore (NIS) route.^8,9^ In contrast to NRPS-dependent pathways, NIS pathways have only recently begun to be biochemically characterized.^10-20^ The interest in NIS pathways has been driven by their novel chemistries and their potential as drug targets in a number of important human pathogens.^21^ The first NIS synthetase to be structurally characterized was the achromobactin biosynthesis protein D (AcsD), from the plant pathogen *Dickeya dadantii* (formally known as *Pectobacterium chrysanthemi*).^20^ AcsD catalyses the enantioselective desymmetrization of citric acid via adenylation of its *pro-R* carboxyl group with ATP. The resulting enzyme-bound homochiral citryl adenylate reacts with l-serine to generate the first committed intermediate, (3′*S*)-*O*-citryl-l-serine, in achromobactin biosynthesis (Fig. 1c). Structural and biochemical studies have characterized the first step of the reaction in detail.^20^ These showed that AcsD is a new member of the adenylating enzyme superfamily, with no sequence or structural homology to other members of the family.^20^

We discovered that AcsD is capable of using other nucleophiles (including amines) in place of l-serine to trap the citryl-adenylate intermediate in the second step of the transformation it mediates.^20^ This has been confirmed by Berti and Thomas, who reported data that are consistent with AcsD-catalyzed ATP-dependent condensation of 1,3-diaminopropane or ethylenediamine with citric acid to yield the corresponding amides.^19^ That the second reaction of AcsD is not limited to simple ester formation with l-serine prompted us to examine the acyl transfer reaction in detail. Here, we report that AcsD is able to use a range of substrates [defining a substrate utilization profile for the acylation (second) step] to assemble amide and ester (and possibly thioester) derivatives of citric acid (Fig. 1d). We have previously demonstrated that the enzyme achieves enantioselective product formation by adenylating only one of the two prochiral carboxyl groups of citric acid.^20^ We assume that the new products, since they must arise from the same chiral intermediate, are likewise single enantiomers. The structure of the AcsD product complex provides a molecular rationale for its substrate recognition profile and allows a catalytic mechanism for the acylation reaction to be proposed. In testing this mechanism by site-directed mutagenesis, we identified a mutant with altered preference (relative to wild type) for amide *versus* ester formation.

## Results

### Substrate utilization profile for acyl transfer reaction of AcsD

**Fig. 2:**
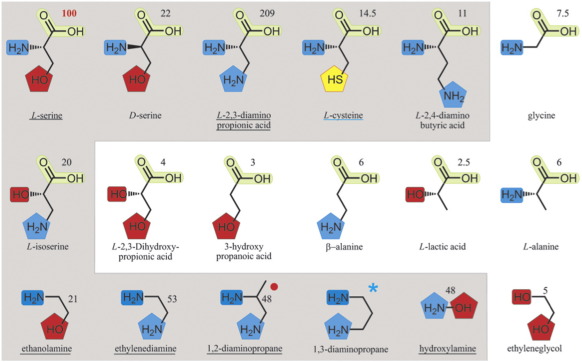
Nucleophile specificity of AcsD: Chemical structures of tested nucleophiles with numbers indicating their percentage reactivity relative to l-serine. Nucleophiles that react twice as fast as water (which has 5% of the reactivity of l-serine) are highlighted in gray and are considered to form a citrate derivative. The original activities with standard deviations are shown in Fig. S1. 2MS, MS/MS, and HRMS analyses were carried out for underlined nucleophiles. Functional groups are highlighted in similar colors for clarity; ★, 1,3-diaminopropane was shown to be active in another study.^19^, mass spectrometric analysis did not resolve which of the amines reacts. The X-ray crystal structure suggests that the primary amine is most likely the nucleophile.

Using a coupled enzyme fluorescence activity assay described previously,^20,22^ we investigated l-serine and 16 other potential substrates (Fig. 2a and Fig. S1) for their ability to promote the conversion of ATP to AMP and pyrophosphate by AcsD in the presence of citric acid. We have previously shown that in the absence of a nucleophile (l-serine), there is still a measurable rate for this reaction, which we attributed to decomposition of the reactive citryl adenylate by water (hydrolysis). We deemed a compound to be a possible substrate if it gave a higher reaction rate than the background hydrolysis. High-resolution mass spectrometry (HRMS) unambiguously confirmed the molecular formulae of the products resulting from the five most reactive nucleophiles (Fig. S2). As previously discussed,^20^ even when the molecular formula is established, there can be ambiguity regarding the arrangement of atoms within the product. Briefly, this arises when there is more than one nucleophilic atom in the acyl acceptor substrate. For example, an oxygen atom (i.e., an alcohol) gives an ester whereas a nitrogen atom (i.e., an amine) gives an amide. l-Serine is one example of where this can arise, because it contains both alcohol and amine groups. We employed tandem mass spectrometry (MS/MS) to resolve any such ambiguities (Fig. S3), having previously established that citryl esters and citryl amides produce distinctive fragmentation patterns.^20^ In the case of l-cysteine, which is a poor substrate, mass spectrometry (MS) identified an ion consistent with citryl-l-cysteine that is only present when enzyme is added (Fig. S4). However, our identification of this product has to remain tentative, because we were unable to obtain HRMS data and, although MS/MS appears to rule out the amide (Fig. S4), there are no fragment ions that can be unambiguously interpreted as arising from the thioester. Our data show that hydroxylamine forms amide and ester products (Figs. S3 and S5). For the other substrates where any such ambiguity can arise, either one or the other product is formed, but not a mixture (within the limits of detection).

In total, 10 substrates (including l-serine) showed activity significantly higher than the background rate of adenylate hydrolysis (Fig. 2). The resulting products include both esters and amides, confirming that AcsD can utilize both oxygen and nitrogen atoms as nucleophiles. Amines are about twice as reactive as the corresponding alcohols, seen by comparing l-2,3-diaminopropionic acid with l-serine, ethanolamine with ethylenediamine, and l-2,3-dihydroxy propionic acid with l-isoserine (in each case, the oxygen atom in the former is replaced by a nitrogen atom in the latter). Examination of the structures of the acyl acceptor substrates for which activity is observed reveals that the most conserved feature is an amino group separated by two intervening carbon atoms from the nucleophilic atom. The three-carbon-atom separation between the amino groups seen in l-2,4-diamino butyric acid results in activity that is only just above background. Substrates lacking an amino group two carbon atoms away from the nucleophile (with the exception of l-isoserine that reverses the oxygen and nitrogen atoms) do not show activity above background. AcsD exhibits a clear preference, but not a requirement, for a carboxylic acid group two carbons from the nucleophile, in the acyl acceptor substrate. This is illustrated by the increased reactivity of l-serine over ethanolamine and l-2,3-diamino propionic acid over ethylenediamine. In the case of serine, the lconfiguration of the carboxylic acid is significantly preferred over the d configuration. Hydroxylamine does not fit this template but hydroxylamine is a very small nucleophilic substrate that, like water, can access the active site.

### Structural studies of AcsD

**Fig. 3:**
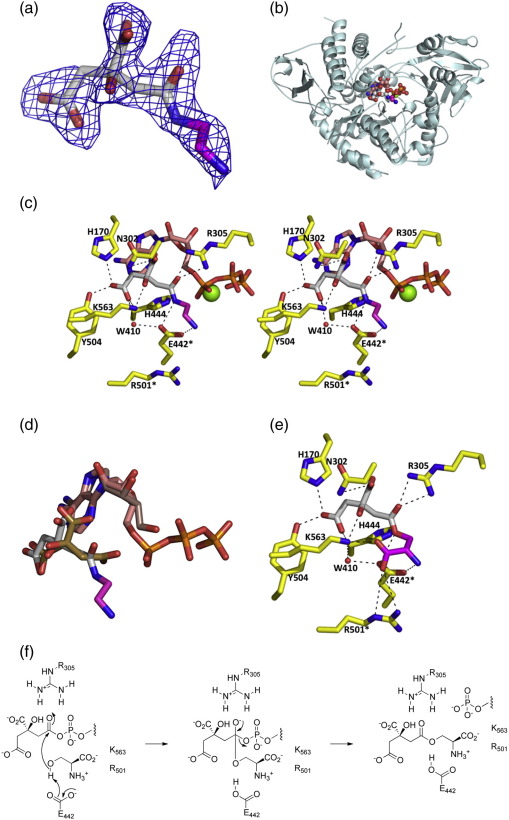
X-ray crystal structure of the AcsD/CEDA co-complex. (a) The *F*_o_ − *F*_c_ electron density map of CEDA at 2.5 σ with a carve radius of 2 Å. This map is derived from phases calculated before any ligand was added to the molecule. The final position of CEDA is shown as sticks with the carbon atoms of citrate colored white, the carbon atoms of ethylenediamine colored magenta, nitrogen atoms colored blue, and oxygen atoms colored red. (b) The protein backbone is shown as a ribbon with monomer B in cyan. CEDA is depicted in a space-filling representation, colored as in (a). ATP is also shown in a space-filling representation, with carbon atoms colored salmon, phosphorus atoms colored orange, and other atoms colored as in (a). Mg^2+^ is shown as a green sphere. (c) Interactions between CEDA and the protein. The side chains of key protein residues are shown in stick representation, with carbon atoms colored yellow and all other atoms colored as in (b). Residues that were mutated in this study are denoted by an asterisk (^⁎^). The H444 and R305 mutants were reported previously.^20^ (d) Superposition of the citrate/adenine (PDB code: 2WO3)^20^ and CEDA/ATP complexes of AcsD. The ligand positions are derived from superposition of the protein atoms. Citrate carbon atoms^20^ are colored olive, and adenine carbon atoms are colored light brown. CEDA carbon atoms are colored white (for those derived from citrate) and magenta (for those derived from ethylenediamine). ATP carbon atoms are colored salmon. The carboxyl group of citrate that undergoes adenylation and subsequent reaction with the hydroxyl group of l-serine changes position significantly in the structures. (e) A model of the *O*-citryl-l-serine complex (made by simple addition of a carboxylate group to the CEDA complex structure). The atoms of l-serine are colored magenta, and other atoms are colored as in (b). The carboxylate group of l-serine is proposed to be recognized by R501 and K563. (f) Proposed mechanism for the reaction of l-serine with the citryl adenylate catalyzed by AcsD.

Attempts to co-crystallize AcsD with *O*-citryl-l-serine or *O*-citryl-ethanolamine generated *in situ* have been unsuccessful, perhaps due to ester degradation. We tried to co-crystallize the enzyme with l-2,3-diamino propionic acid (again generated *in situ*), but this failed to give clear density for a new complex. However, growing crystals of AcsD pre-incubated with ATP, Mg^2+^, and ethylenediamine showed clear additional electron density for a molecule larger than citric acid in one subunit as well as density for ATP and Mg^2+^ (Fig. 3a). We interpret this density as (3′*S*)-*N*-citryl-ethylenediamine (CEDA) and were able to refine the co-complex (Fig. 3b and c). Data collection and refinement statistics are shown in Table 1. We do not have an explanation for why this density is found in only one subunit. ATP, Mg^2+^, the protein, and much of the citryl moiety of CEDA are in the same positions they have been previously observed to occupy in other co-complexes (Fig. 3d). We attribute the observation that ATP (rather than AMP) is bound to be due to the large excess of ATP in the solution. The difference in position between the citryl moiety of CEDA and citrate in the previously reported citrate/adenosine/sulfate complex^20^ occurs in the regions occupied by the carboxamide group in CEDA (this work) and the *pro-R* carboxyl group of citrate (previous structure^20^). The carboxamide group adopts a rotamer (Fig. 3d) different from that of the citrate carboxyl group. If the carboxamide group did not do this, the ethylenediamine moiety would clash with the α-phosphate of ATP (and AMP). The ethylenediamine moiety of CEDA sits in a shallow cleft and makes several contacts with the protein. There is a particularly striking bidentate hydrogen bond interaction between both nitrogen atoms of CEDA and the two oxygen atoms of the side chain of E442 (Fig. 3c). The side chains of R501 and K563 point towards the ethylenediamine moiety. Using the atoms of CEDA, we constructed a model of the cognate *O*-citryl-l-serine product complex (by addition of an l-configured carboxylate group and changing the amide to an ester). In this model, the carboxylate group interacts with K563 and R501 (Fig. 3f).

### Site-directed mutagenesis studies

**Fig. 4:**
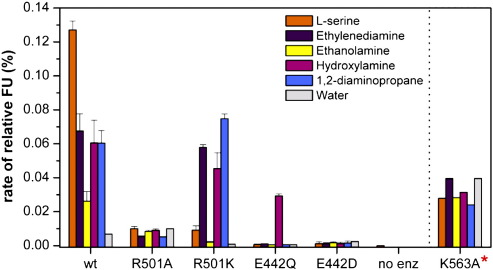
Mutation of residues predicted to be important in recognition and catalysis of the second step of the transformation mediated by AcsD. The specific activity is shown as fluorescence units (FU) on the *y*-axis. The data for the K563A mutant overestimate the specific activity, because the protein was not fully purified due to low levels of expression. The R501K mutant shows a reversal in specificity. It is more active with ethylenediamine than the wild-type enzyme and less active with the cognate substrate l-serine.

We decided to probe the roles of R501, E442, and K563 in substrate recognition and catalysis by engineering the following mutations: R501A, R501K, K563A, E442D, and E442Q. Each mutant was confirmed as folded and tested for activity with l-serine, ethylenediamine, ethanolamine, 1,2-diaminopropane, and hydroxylamine. The E442D and R501A mutants were inactive with all nucleophiles tested (Fig. 4). The E442Q mutant was inactive with all nucleophiles except hydroxylamine, with which a reduced level of activity was retained. The R501K mutant retained approximately the same level of activity with ethylenediamine and hydroxylamine, 14-fold less activity with l-serine, and 20% more activity with 1,2-diaminopropane, compared to wild-type AcsD. The K563A mutant was very poorly expressed, and samples always contained contaminating proteins. The mutant had reduced but real activity, although accurate quantitation of the specific activity was not possible.

## Discussion

AcsD catalyzes the biosynthesis of *O*-citryl-l-serine from citrate and l-serine in two chemically and temporally distinct steps.^20^ An enzyme-bound reactive citryl adenylate is the product of the first step and the substrate for the second step, which utilizes l-serine. l-Serine and a number of other substrates are utilized significantly faster in the second (acyl transfer) step than the competing hydrolysis of the citryl adenylate. We interpret this as evidence that the acyl transfer reaction involves both substrate recognition and catalysis. The ability to catalyze two successive reactions is a general feature of adenylating enzymes, which are known to make thioesters, esters, and amides via activated adenylate intermediates.^23^ In other members of the superfamily, the switch between the two steps is accompanied by a large conformation change.^24,25^ The structure of the product complex presented here suggests that no such large rearrangement occurs in AcsD.

Both ethylenediamine and 1,3-diaminopropane appear to be capable of replacing l-serine in the second step of the AcsD-catalyzed reaction,^19^ establishing that the enzyme can make both amides and esters.^20^ There is only one other report of an adenylating enzyme that can catalyze formation of two different types of bond (thioester and amide) with the adenylate intermediate bound within the active site.^26^ The ability of an NRPS thioesterase domain to catalyze formation of both amides and esters within the same active site has recently been demonstrated.^27^ AcsD utilizes substrates with a nitrogen atom as nucleophile more readily than those with oxygen. Unusually then, AcsD seems able to catalyze formation of esters and amides from a common citryl-adenylate intermediate. AcsD may also be capable of catalyzing formation of a thioester with l-cysteine.

The preference of AcsD for amine nucleophiles poses the question: how does the enzyme select against the amino groups within amino acids and other amines in the biological milieu? Our data indicate that this is accomplished by imposing a recognition requirement for an amino group separated by two intervening carbon atoms from the nucleophilic atom (N, O, or S). Structural data show that AcsD uses E442 to achieve this recognition by making a striking bidentate interaction with CEDA. Consistent with the importance of this interaction for substrate recognition, the mutation E442D abolishes activity with all substrates. AcsD also favors an l-configured carboxylate group (also two carbons from the nucleophilic atom), and a model of the *O*-citryl-l-serine AcsD complex suggests that the side chains of K563 and R501 underpin this recognition. The R501K mutant, which preserves both a positive charge and a long side chain, retains its activity with l-serine and l-2,3-diaminopropionic acid (Fig. S6) (but at reduced rates). Ethylenediamine and 1,2-diaminopropane, which lack carboxylate groups, are utilized at or above native rates by the R501K mutant. These data are consistent with R501 playing a role in the recognition of the l-configured carboxylate group. Our analysis of the K563A mutant is hindered by our inability to purify it fully due to its low overexpression level, but it does appear to have reduced activity. However, K563 also contacts citric acid, and its mutation is likely to affect both the first and second steps of the AcsD-catalyzed reaction.

l-Isoserine illustrates where the balance between recognition and reactivity lies. It uniquely has a nitrogen atom as the nucleophile and an l-configured carboxylate group, but it has a hydroxyl group, rather than an amino group, two carbons from the nucleophilic atom. This compound is barely active, and it is the only compound with a hydroxyl group two carbons from the nucleophilic atom where we detected any activity. Thus, we conclude that it is recognition of an amino group two carbons from the nucleophilic atom that lies at the heart of the ability of AcsD to select the desired substrate from the biological milieu. This is augmented by a preference for an l-configured carboxylate. Only l-cysteine and l-serine among common biological molecules fulfill these requirements. We rationalize the apparently slow reaction with l-cysteine by the large S atom disrupting the interaction with E442, which in turn perturbs the active site.

Besides rationalizing the recognition of the acyl acceptor substrate, the data illuminate the catalytic mechanism, particularly the role of E442. The carboxylate side chain of this residue is ideally positioned to enhance the reactivity of the nucleophilic nitrogen or oxygen atom by acting as a general base (Fig. 3f). Mutation of this residue to Q abolishes activity for all substrates (except hydroxylamine) and seems to reduce the background rate for water, consistent with our proposed role for E422. Hydroxylamine is a potent nucleophile with a p*K*_a_ < 8 and, as consequence, is not expected to require deprotonation by a general base. A conserved general base has not been identified in enzymes belonging to the acyl-adenylate-forming superfamily, and in the structure of the thioester-forming conformation of human medium-chain acyl-coenzyme A synthetase (ACSM2A), no equivalent residue can be found.^28^ In the thiol-forming conformation of the adenylating enzyme DltA, D196 is close to the active site, but its role was proposed to be limited only to recognizing the amino group of d-alanine.^29^ Even at pH 7, a significant proportion of thiol (p*K*_a_ ∼ 8) will be found in the deprotonated (thiolate) form; thus, a general base may not be required for thioester formation in DltA. R305 is crucial for adenylate formation,^20^ and our model suggests that it also stabilizes the tetrahedral intermediate formed by the attack of l-serine on the citryl-adenylate intermediate (Fig. 3f).

For substrates that lack a carboxylate group, we note that the R501K mutant has essentially wild-type activity, whereas the R501A mutant is inactive. If R501 were to play a role in substrate recognition only, one might expect both these mutants to behave in similar ways with substrates lacking carboxylate groups. That this is not true leads us to consider a role for R501 in catalysis. The CEDA/AcsD co-complex structure shows R501 to be distant from the site of nucleophilic attack, and a direct role in catalysis is therefore unlikely. Our favored explanation is that the aliphatic side chain of R501 is important for correct positioning of the side chain of the E442 general base (Fig. 3c).

R501K reverses the wild-type enzyme's preference for ester formation with l-serine over amide formation with 1,2-diaminopropane (from 2:1 to 1:7). Hydrolysis of the citryl adenylate competes with acyl transfer in AcsD-catalyzed reactions. Therefore, for a change in specificity to be useful in synthesis, it must not be accompanied by a loss of activity; otherwise, the rate of desired product formation relative to competing hydrolysis would be too low. R501K meets this test as it has an enhanced utilization (relative to wild-type enzyme) of 1,2-diaminopropane. We attribute this to the greater volume in the active site of the R501K mutant (resulting from loss of the guanidinium group). This permits the hydrophobic CH_3_ group of 1,2-diaminopropane to bind without disrupting the orientation of the catalytic residues.

## Summary

A clear understanding of the molecular mechanisms for substrate recognition and catalysis by NIS synthetases could aid the development of inhibitors for this important family of enzymes. Additionally, our data show that AcsD is capable of enantioselective desymmetrization of citric acid using a wide range of nucleophiles, and we have developed a clear set of rules for substrate recognition. We have also been able to create a mutant enzyme with an altered preference for substrates, indicating that AcsD and related enzymes may find future application in stereoselective synthesis.

## Experimental Procedures

### Mutagenesis

AcsD mutants containing R501A and R501K were constructed using the QuikChange mutagenesis kit (Stratagene) with the following primers: 5′CGAACAGGGCTGGAAT**GCC**ATCATGTACTGCCTG3′ (R501A forward), 5′CAGGCAGTACATGAT**GGC**ATTCCAGCCCTGTTCG3′ (R501A reverse), 5′CCGCGAACAGGGCTGGAAT**AAA**ATCATGTACTGCCTGTTC3′ (R501K forward), and 5′GAACAGGCAGTACATGAT**TTT**ATTCCAGCCCTGTTCGCGG3′ (R501K reverse). Mutants containing E442Q, E442D, and K563A were constructed using a modified QuikChange (Stratagene) procedure^26^ with the following primers:

5′GCGTGGTGATG**CAG**CCGCACCTGCAAAACAGCGTG3′ (E442Q forward), 5′GG**CTG**CATCACCACGCCGTGATTGAAGAACAGCGAC3′ (E442Q reverse), 5′GCGTGGTGATG**GAT**CCGCACCTGCAAAACAGCGTG3′ (E442D forward), 5′GG**ATC**CATCACCACGCCGTGATTGAAGAACAGCGAC3′ (E442D reverse), 5′CGGTAGCGTGC**GCG**ACCAACCTTAAGGTCCGGCTGG3′ (K563A forward), and 5′AGGT**CGC**GCACGCTACCGGATGGCCGGCGATCAGC3′ (K563A reverse). Possible wobble base pairs were avoided during primer design. All generated mutations were verified by DNA sequencing.

### Overproduction, purification, and co-crystallization with ethylenediamine

Expression, purification, tag removal, and crystallization of the wild-type protein followed established procedures.^30–32^ AcsD has six additional residues at the N-terminus (GIDPFT) after tag removal. Mutant proteins for assays were purified by a single 5-mL Ni-affinity column (GE Healthcare); dialyzed in 50 mM Tris–HCl, pH 7.5, 500 mM NaCl, and 10% (v/v) glycerol; and concentrated to a final concentration of 4–8 mg/mL.

For co-crystallization with CEDA, 9 mg/mL AcsD [stored in protein buffer: 50 mM Tris–HCl, pH 7.5, 500 mM NaCl, and 10% (v/v) glycerol] was incubated for 1 h with 15 mM ethylenediamine, 10 mM MgCl_2_, 15 mM ATP, and 15 mM citrate. The supernatant was used to grow CEDA co-complex crystals in hanging drops from equal mixtures with 0.1 M Hepes, pH 7.2, 17% (w/v) polyethylene glycol 8000, and 7.5% (v/v) glycerol at 293 K. Data were collected from a single frozen crystal. The AcsD native structure [Protein Data Bank (PDB) code: 3FFE] was used to phase the 2-Å data of the CEDA/ATP co-complex. The structure was refined using REFMAC5,^33,34^ and Coot^35^ was used for manual manipulation. The ligand and the library were built using the PRODRG server.^36^ We were unable to locate residues 1–2 and 589–626 for monomer A and residues 1–3, 573–577, and 588–626 for monomer B in the experimental maps. While ATP is present in both monomers, CEDA has only clear density in monomer B (Fig. 3b). Data collection and refinement statistics are presented in Table 1, and structural data are deposited with PDB code 2x3j.

### AMP production assay

The assay is based on coupling the AcsD-dependent formation of AMP to the lactate dehydrogenase oxidation of NADH,^22^ with NADH concentration monitored in real time by fluorescence (excitation, 376 nm; emission, 462 nm), and has been described in detail elsewhere.^20^ The reaction contained 50 mM Tris–HCl buffer (pH 8.0), 3 mM ATP, 15 mM MgCl_2_, 1.5 mM phosphoenolpyruvate, 0.25 mM NADH, 2 μM His_6_-AcsD, 12.6 U of lactate dehydrogenase, 8.4 U of pyruvate kinase, 4 U of myokinase, 75 mM nucleophile, and 2 mM citric acid at a total volume of 140 μL. The mixture was incubated at 293 K for 5 min, and the reaction was started by the addition of wild-type or mutant enzyme (2 μM).

### MS and MS/MS analysis

The enzymatic reaction was carried out in ammonium acetate buffer (10 mM, pH 8.0) containing 10–30 μM AcsD, 30 mM MgCl_2_, 20 mM citrate, and 40 mM of various nucleophiles (each buffered at pH 8). The mixture was incubated at 20 °C, and the reaction was initiated by the addition of 20 mM ATP. At defined time points, 1-μL aliquots were removed, diluted into 50:50 acetonitrile:water (100 μL), and centrifuged at 12,000 rpm for 1 min. The samples were analyzed using continuous syringe infusion (Harvard syringe pump, Harvard Apparatus, Kent, UK) into an LCT (Micromass, Manchester, UK) ESI MS instrument or a Q-Star XL (Applied Biosystems, Foster City, CA) ESI Q-TOF instrument using the appropriate ionization mode. *m*/*z* values of interest were subjected to collisionally induced dissociation on the Q-Star XL instrument, with collision energy adjustment to give some intact signal remaining.

All HRMS measurements were carried out on a Bruker MaXis mass spectrometer in positive and negative ion modes by direct infusion at 2 μL/min. The scan range was 50–2000 *m*/*z*, and calibration was done by direct infusion of 10 mM sodium formate before each measurement. The capillary voltage was set at − 3000 V (positive mode) or + 4000 V (negative mode), the nebulizer gas was at 0.4 bar, the dry gas flow was 4 L/min, and the dry temperature was 180 °C. Ion transfer parameters were as follows: funnel RF, 200 Vpp; multiple RF, 200 Vpp; quadrupole low mass, 55 *m*/*z*; collision energy, 5 ev; collision RF, 600 Vpp; ion cooler RF ramping from 50 to 250 Vpp; transfer time, 121 μs; and pre-pulse storage time, 1 μs.

### Accession number

Data collection and refinement statistics are presented in Table 1, and structural data are deposited with PDB code 2x3j.

## Figures and Tables

**Table 1:** X-ray data collection and refinement statistics

	CEDA complex
*Data collection*	
Space group	*P*1
Cell dimensions	
*a*, *b*, *c* (Å)	57.7, 71.5, 95.6
α, β, γ (°)	97.3, 102.0, 91.0
Resolution (highest shell) (Å)	45–2.00 (2.07–2.00)
*R*_merge_	0.064 (0.350)
*I*/σ*I*	12.8 (2.7)
Completeness (%)	93.6 (73.4)
Redundancy	3.1 (2.1)
	
*Refinement*	
No. of reflections	93,717
*R*_work_/*R*_free_	0.175/0.206
No. of atoms	
- Protein	9462
- CEDA	16
- Water	597
- ATP	62
- Mg^2+^	2
- Glycerol	30
*B*-factors	
- Protein	48
- CEDA	58
- Water	50
- ATP	48
- Mg^2+^	52
- Glycerol	40
RMSD	
Bond lengths (Å)	0.009
Bond angles (°)	1.12
Ramachandran	
Allowed/forbidden	97.9/0.2
MolProbity score/centile	1.34/99
PDB code	2x3j
