# Supplementary material for: Structural Basis for Acyl Acceptor Specificity in the Achromobactin Biosynthetic Enzyme AcsD
Source: J Mol Biol. 2011 Sep 23;412(3):495–504. doi: 10.1016/j.jmb.2011.07.059 (PMC3323832; doi:10.1016/j.jmb.2011.07.059)
Supplement: Supplementary materials [file mmc1.pdf]

Structural basis for acyl acceptor specificity in the achrombactin biosynthetic enzyme AcsD

Stefan Schmelz[1], Catherine H. Botting[1], Lijiang Song[2], Nadia Kadi[2], Gregory L. Challis[2], James H. Naismith[1*]*

[1] Scottish Structural Proteomics Facility and Centre for Biomolecular Sciences, The University, St Andrews, Scotland, U.K. KY16 9ST

[2] Department of Chemistry, University of Warwick, Coventry, U.K. CV4 7AL

* = corresponding authors: naismith@st-and.ac.uk; g.l.challis@warwick.ac.uk

Supporting Information

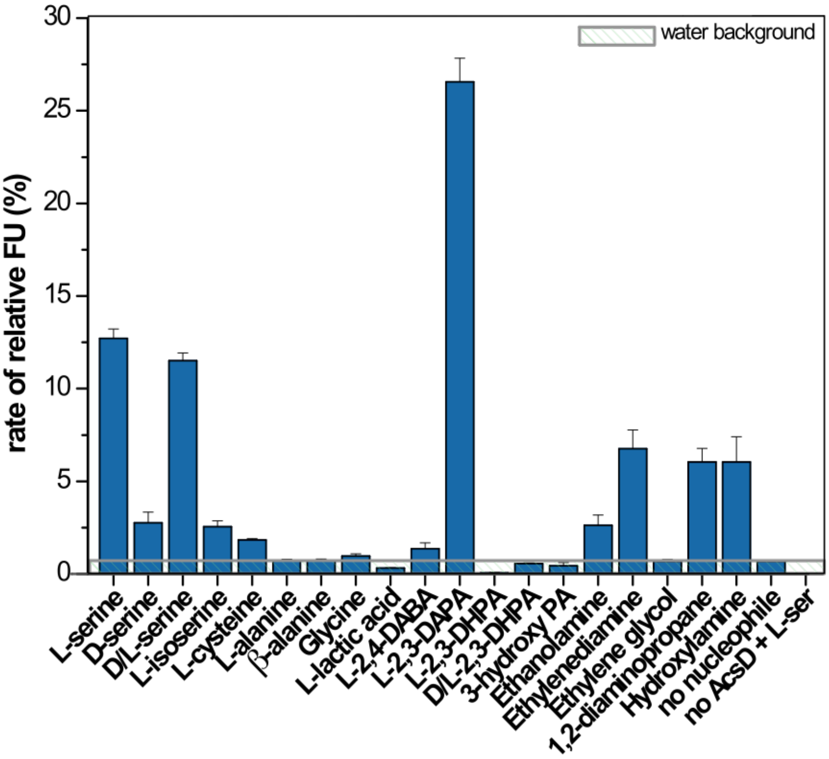

Figure S1: Results of the AcsD activity assay for the following nucleophiles: L-serine, D-serine, D/L-serine, L-isoserine, L-cysteine, L-alanine, glycine, L-lactic acid, L-2,4-diamino butyric acid (L-2,4-DABA), L-2,3-diamino propionic acid (L-2,3-DAPA), L-2,3-dihydroxy propionic acid (L-2,3-DHPA), D/L-2,3-dihydroxy propionic acid (D/L-2,3-DHPA), 3-hydroxy propanoic acid (3-hydroxy PA), ethanolamine, ethylenediamine, ethylene glycol, 1,2-diaminopropane, hydroxylamine, no nucleophile (except water) and a control lacking AcsD. Error bars (one standard deviation) are shown and are derived from three independent experiments.

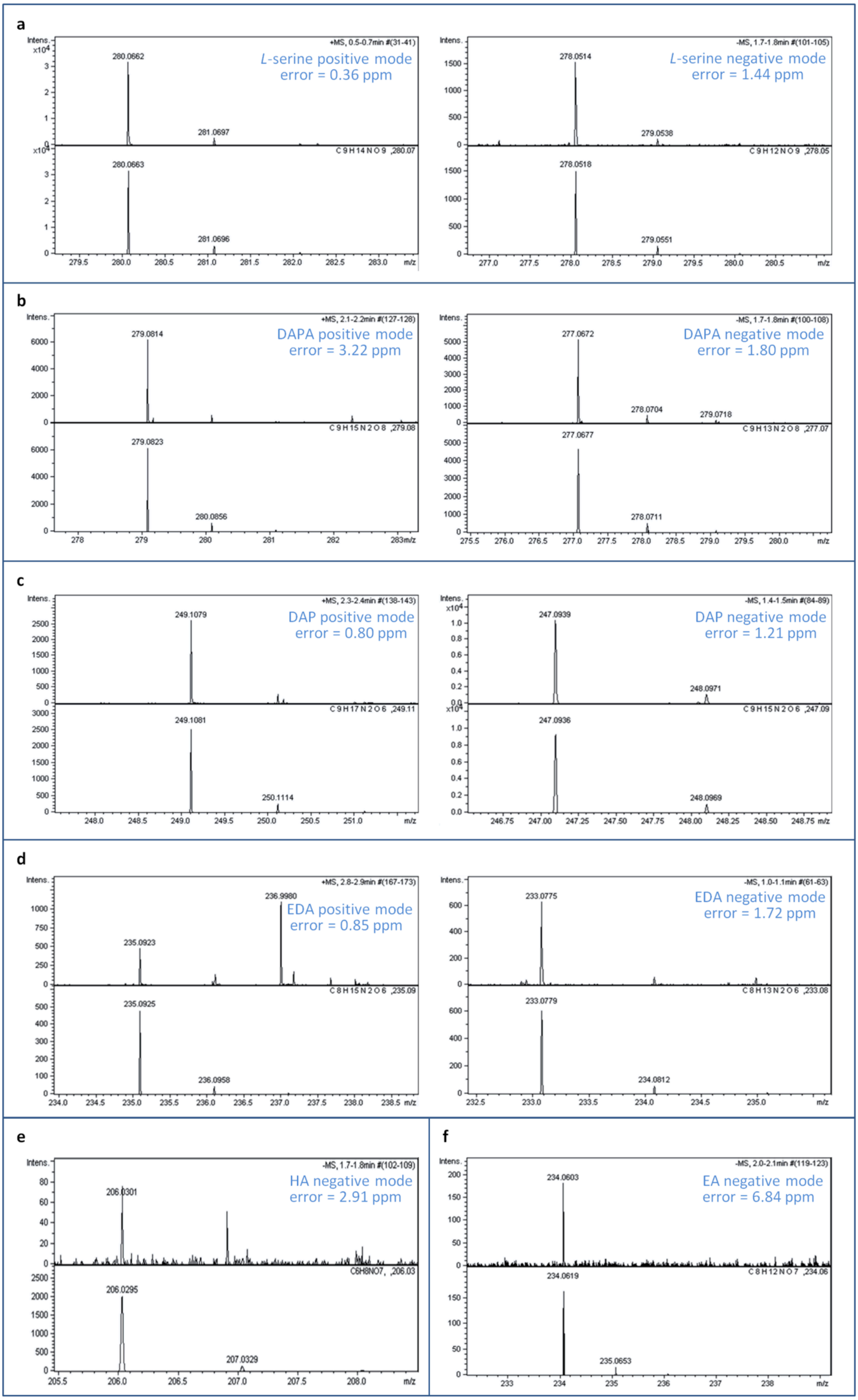
a
+MS, 0.5-0.7min #(31-41)
L-serine positive mode
error = 0.36 ppm
280.0662
281.0697
C9H14NO9 ,280.07
280.0663
281.0696
-MS, 1.7-1.8min #(101-105)
L-serine negative mode
error = 1.44 ppm
278.0514
279.0538
C9H12NO9 ,278.05
278.0518
279.0551
b
+MS, 2.1-2.2min #(127-128)
DAPA positive mode
error = 3.22 ppm
279.0814
C9H15N2O8 ,279.08
279.0823
280.0856
-MS, 1.7-1.8min #(100-108)
DAPA negative mode
error = 1.80 ppm
277.0672
278.0704
279.0718
C9H13N2O8 ,277.07
277.0677
278.0711
c
+MS, 2.3-2.4min #(138-143)
DAP positive mode
error = 0.80 ppm
249.1079
C9H17N2O6 ,249.11
249.1081
250.1114
-MS, 1.4-1.5min #(84-89)
DAP negative mode
error = 1.21 ppm
247.0939
248.0971
C9H15N2O6 ,247.09
247.0936
248.0969
d
+MS, 2.8-2.9min #(167-173)
EDA positive mode
error = 0.85 ppm
236.9980
235.0923
C8H15N2O6 ,235.09
235.0925
236.0958
-MS, 1.0-1.1min #(61-63)
EDA negative mode
error = 1.72 ppm
233.0775
C8H13N2O6 ,233.08
233.0779
234.0812
e
-MS, 1.7-1.8min #(102-109)
HA negative mode
error = 2.91 ppm
206.0301
C6H8NO7, ,206.03
206.0295
207.0329
f
-MS, 2.0-2.1min #(119-123)
EA negative mode
error = 6.84 ppm
234.0603
C8H12NO7 ,234.06
234.0619
235.0653
Intens.
m/z

Figure S2: High resolution mass spectrometry of AcsD ester and amide products. **a** Observed (top row) and calculated mass (bottom row) of the AcsD product with L-serine as nucleophile in positive (left) and negative (right) ionization mode. **b** Observed (top row) and calculated mass (bottom row) of the AcsD product with L-2,3-diamino propionic acid (DAPA) as nucleophile in positive (left) and negative (right) ionization mode. **c** Observed (top row) and calculated mass (bottom row) of the AcsD product with 1,2-diaminopropane (DAP) as nucleophile in positive (left) and negative (right) ionization mode. **d** Observed (top row) and calculated mass (bottom row) of the AcsD product with ethylenediamine (EDA) as nucleophile in positive (left) and negative (right) ionization mode. **e** Observed (top row) and calculated mass (bottom row) of the AcsD product with hydroxylamine (HA) as nucleophile in negative ionization mode. **f** Observed (top row) and calculated mass (bottom row) of the AcsD product with ethanolamine (EA) as nucleophile in negative ionization mode. Note that the last two spectra (e and f) are noisier due to their lower activity as AcsD nucleophiles compared to DAPA or L-serine.

a

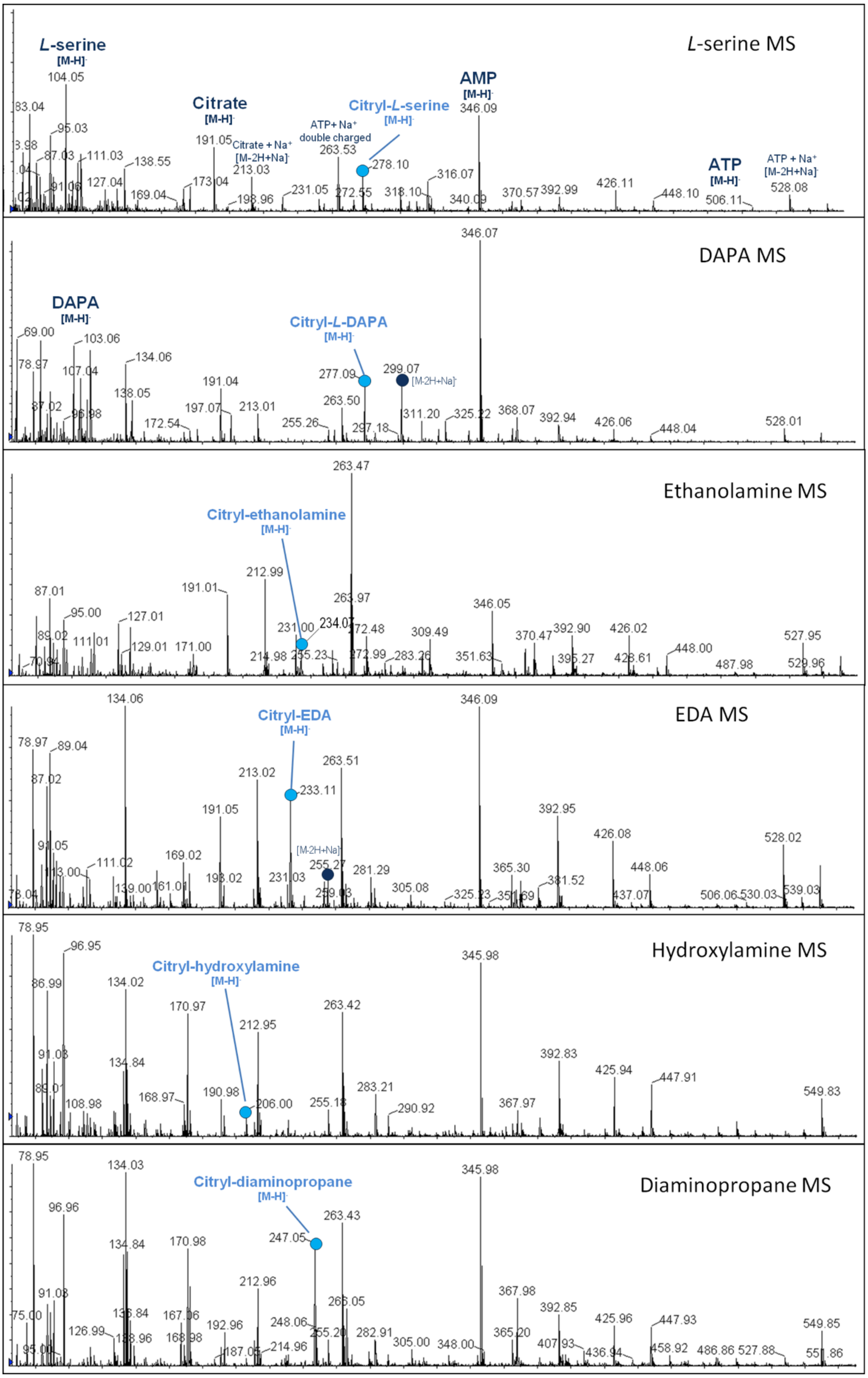
L-serine MS
L-serine
[M-H]-
Citrate
[M-H]-
Citrate + Na+
[M-2H+Na]-
ATP+ Na+
double charged
Citryl-L-serine
[M-H]-
AMP
[M-H]-
ATP
[M-H]-
ATP + Na+
[M-2H+Na]-
DAPA MS
DAPA
[M-H]-
Citryl-L-DAPA
[M-H]-
[M-2H+Na]-
Ethanolamine MS
Citryl-ethanolamine
[M-H]-
EDA MS
Citryl-EDA
[M-H]-
[M-2H+Na]-
Hydroxylamine MS
Citryl-hydroxylamine
[M-H]-
Diaminopropane MS
Citryl-diaminopropane
[M-H]-

b

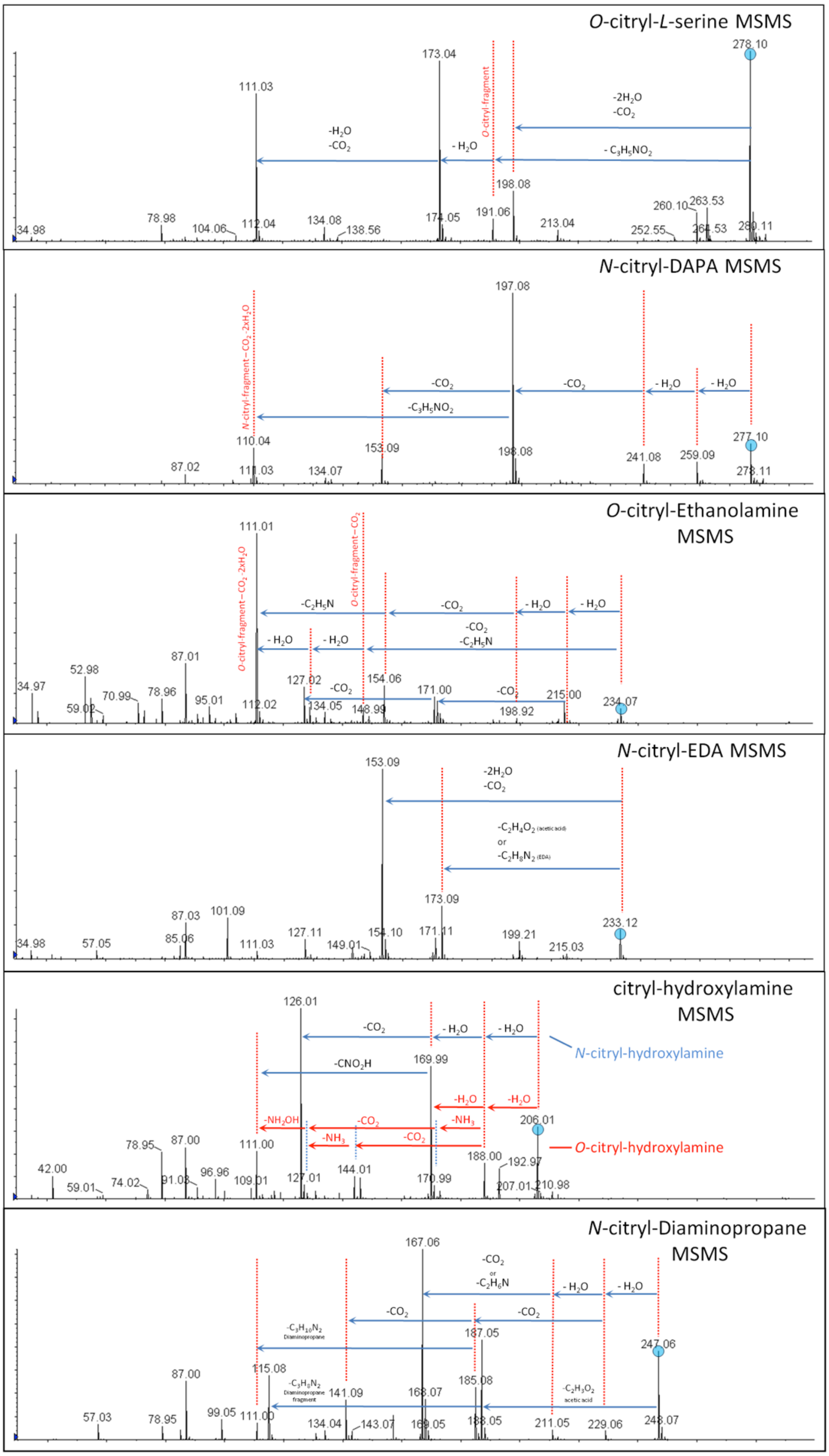
O-citryl-L-serine MSMS
N-citryl-DAPA MSMS
O-citryl-Ethanolamine MSMS
N-citryl-EDA MSMS
citryl-hydroxylamine MSMS
N-citryl-hydroxylamine
O-citryl-hydroxylamine
N-citryl-Diaminopropane MSMS

Figure S3: MS and MS/MS analysis of AcsD catalyzed citric acid ester and amid. **a** MS reaction analysis on the Q-Star XL instrument. In all tested reactions production of AMP (*m/z* =346 $[M-H]^-$) was observed, while ATP (*m/z* =506 $[M-H]^-$ and *m/z* =528 $[M-2H+Na]^-$) was consumed. Respective product peaks were marked with light blue circles. For citryl-L-DAPA or citryl-EDA a second peak corresponding to *m/z*= $[M-2H+Na]^-$ (dark blue circle) could be assigned. **b** MS/MS analysis of AcsD catalyzed products on the Q-Star XL instrument. Respective product peaks are labeled with light blue circles with mass losses to due fragmentation assigned. Fragmentation pattern including lost fragments and masses were assigned. In the case of hydroxylamine daughter fragments corresponding to N-citryl-hydroxylamine (blue) or O-citryl-hydroxylamine (red) were identified.

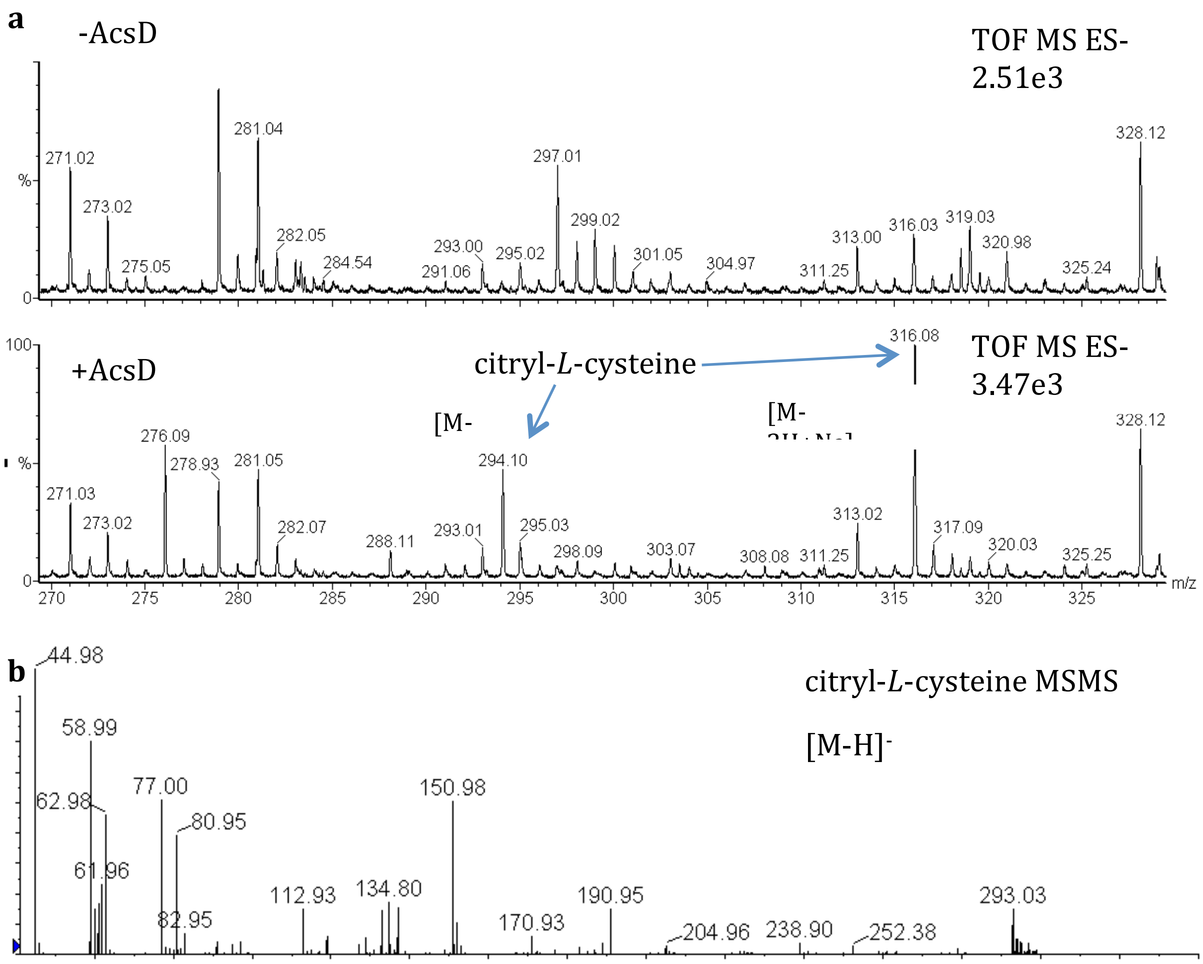

Figure S4: MS analysis of AcsD reaction with L-cysteine **a** Only in reactions where AcsD was present was a new peak with a m/z value of 294.11 observed on the LCT (Micromass, Manchester, UK) ESI MS instrument. This was observed in multiple repeats of the experiment. This peak corresponds to the theoretical m/z= 294.03 $[M-H]^-$ value of citryl-L-cysteine. **b** MS/MS analysis of AcsD catalyzed products on the Q-Star XL instrument. The peak at m/z 111 characteristic for amide formation is absent [1]. The mass at 150.98 matches $C_6HSO_3^-$ (loss of 3 waters and $C_3H_5NO_2$ (main chain of amino acid)). We were unable to obtain definitive fragmentation data and our assignment of the citryl-L-cysteine remains tentative.

a

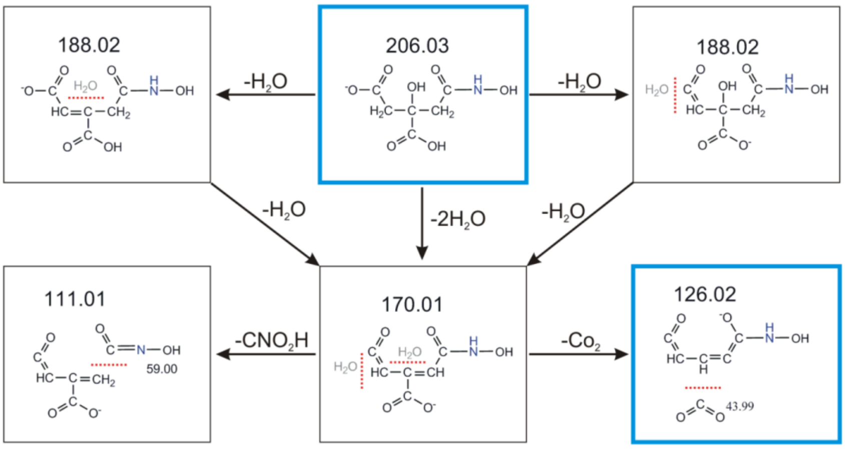

b

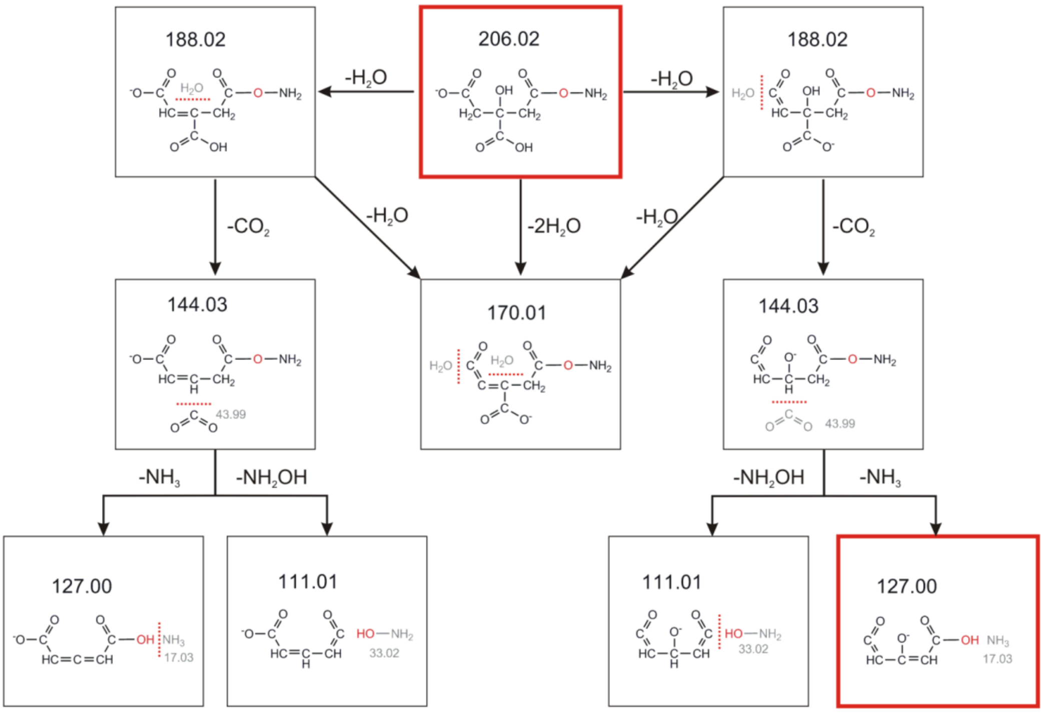

Figure S5: Theoretical fragmentation pattern of citryl-hydroxylamine a) Possible fragments for N-citryl-hydroxylamine b) Possible fragments for O-citryl-hydroxylamine. Peaks for both are observed Figure S3.

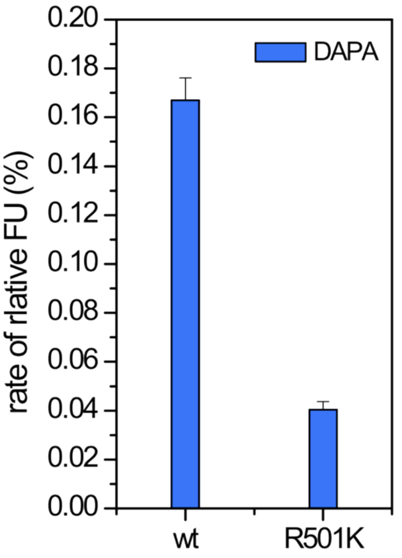

Figure S6: Activity assay of wild type AcsD and a R501K mutant with 2,3-diaminopropionic acid (DAPA).

(1) Schmelz, S.; Kadi, N.; McMahon, S. A.; Song, L.; Oves-Costales, D.; Oke, M.; Liu, H.; Johnson, K. A.; Carter, L. G.; Botting, C. H.; White, M. F.; Challis, G. L.; Naismith, J. H. *Nat Chem Biol* **2009**, *5*, 174-182.
